# Wideband-Switchable Metamaterial Absorber Using Injected Liquid Metal

**DOI:** 10.1038/srep31823

**Published:** 2016-08-22

**Authors:** Hyung Ki Kim, Dongju Lee, Sungjoon Lim

**Affiliations:** 1 School of Electrical and Electronic Engineering, Chung-Ang University, Heukseok-Dong, Dongjak-Gu 156-756, Republic of Korea

## Abstract

Metamaterial absorbers can provide good solutions for radar-cross-section (RCS) reduction. In spite of their attractive features of thinness, lightness, and low cost, resonant metamaterial absorbers have a drawback of narrow bandwidth. For practical radar applications, wideband absorbers are necessary. In this paper, we propose a wideband-switchable metamaterial absorber using liquid metal. In order to reduce RCS both for X-band and C-band, the switchable Jerusalem cross (JC) resonator is introduced. The JC resonator consists of slotted circular rings, chip resistors, and microfluidic channels. The JC resonator is etched on a flexible printed circuit board (FPCB), and the microfluidic channels are laser-etched on a polydimethylsiloxane (PDMS) material. The proposed absorber can switch the absorption frequency band by injecting a liquid metal alloy into the channels. The performance of the absorber was demonstrated through full-wave simulation and through measurements employing prototypes. The experimental results showed absorption ratios of over 90% from 7.43 GHz to 14.34 GHz, and from 5.62 GHz to 7.3 GHz, with empty channels and liquid metal-filled channels, respectively. Therefore, the absorption band was successfully switched between the C-band (4–8 GHz) and the X-band (8–12 GHz) by injecting liquid metal eutectic gallium indium alloy (EGaIn) into the channels.

There have been various attempts to develop low-observable technologies and reduce radar cross section (RCS), such as stealth technology. An EM wave absorber can minimize reflection and transmission of EM waves, and can therefore be used for preventing EM interference^1^ and in realizing stealth technologies^2^.

Metamaterial-based EM wave absorbers have been widely researched because of their low profile and simple fabrication process. The metamaterial’s permittivity and permeability can be artificially manipulated by employing EM resonators, such as split ring resonators (SRRs)^3^. By using the characteristics of a metamaterial, a metamaterial absorber can achieve impedance matching with free space. The metamaterial absorber shows an almost perfect absorption rate; however, it suffers from narrow bandwidth because its absorption is based on electric and magnetic resonances. In order to overcome the narrow bandwidth of metamaterial absorbers, several approaches have been proposed. Generating multi-resonance is one approach to improving the bandwidth^4^,^5^,^6^. However, it is difficult to handle all resonant frequencies simultaneously; and it would still be insufficient to cover the entire X-band. Another approach for achieving wide bandwidth is that of using a lossy pattern, implemented by printing with resistive ink^7^,^8^,^9^,^10^. It does produce a wide bandwidth; however, it is hard to gain the performance predicted by EM simulation tools due to non-uniform ink distribution. An ultra-wideband absorber has also been proposed^11^ through the use of a multi-layer board and lumped components. It consists of three layers: an artificial impedance surface (AIS) layer, an air layer, and a resistor-capacitor (RC) layer. It covers the entire X-band. However, it is hard to maintain a uniform thickness for the air layer as the absorber becomes larger. A frequency-tuneable metamaterial absorber can be a substitute for bandwidth limitation. By using active components or materials, such as diodes^12^,^13^, micro-electromechanical systems (MEMS)^14^, liquid crystals^15^, and vanadium oxide^16^, the absorption frequency of a metamaterial absorber can be adjusted. In addition, it is difficult to reduce RCS both in X-band and C-band with a single wideband metamaterial absorbers. Therefore, a frequency-switchable absorber can be a solution to cover multiple frequency bands.

Metamaterial absorbers are generally realized on hard substrates, such as FR4 material^3^,^4^,^6^,^11^,^12^,^13^,^17^, vanadium oxide^16^, and silicon^14^,^18^. These hard materials are inflexible, and can therefore be applied only on a planar surface. In order to allow flexibility, a thin film substrate can be adapted for fabrication of a metamaterial absorber^2^,^19^,^20^,^21^. Therefore, flexible metamaterial absorbers are necessarily for both planar and curve surfaces.

In the present study, we introduce a flexible wideband-switchable metamaterial absorber by integrating lumped components and microfluidic channels. The microfluidic channel is used as a switching component. The switching capability of liquid metal for the switchable metamaterial absorber was previously reported with 1-dimensional structure^22^. It shows drawbacks of narrow bandwidth and limited switching range. In this work, we proposed the novel switchable 2-dimensional periodic structure on flexible materials. The proposed absorber can switch the absorption frequency band by injecting a liquid metal alloy into microfluidic channels while keeping wideband in X-band. We used eutectic gallium-indium (EGaIn: 75% Ga, 25% In, by weight) to fill the microfluidic channels^23^. When EGaIn is exposed to air, it generates a thin oxide layer on its surface. This oxide layer prevents evaporation, improves mechanical stability, and maintains EGaIn’s performance. It has been also reported that EGaIn can be used in THz although its DC conductivity is an order of magnitude smaller than other metals^24^. The proposed absorber is composed of a Jerusalem cross (JC) resonator with slotted circular ring, and with chip resistors being used to increase the bandwidth. The JC resonator is etched on a flexible printed circuit board (FPCB) substrate using conventional PCB manufacturing processes. The microfluidic channels are laser-etched on a flexible polydimethylsiloxane (PDMS) substrate. Therefore, the proposed absorber has the advantage of flexibility. The performance of the proposed absorber was investigated through full-wave simulation and through measurements employing prototypes.

## Results

### Sample schematic and fabricated prototype

**Figure 1:**
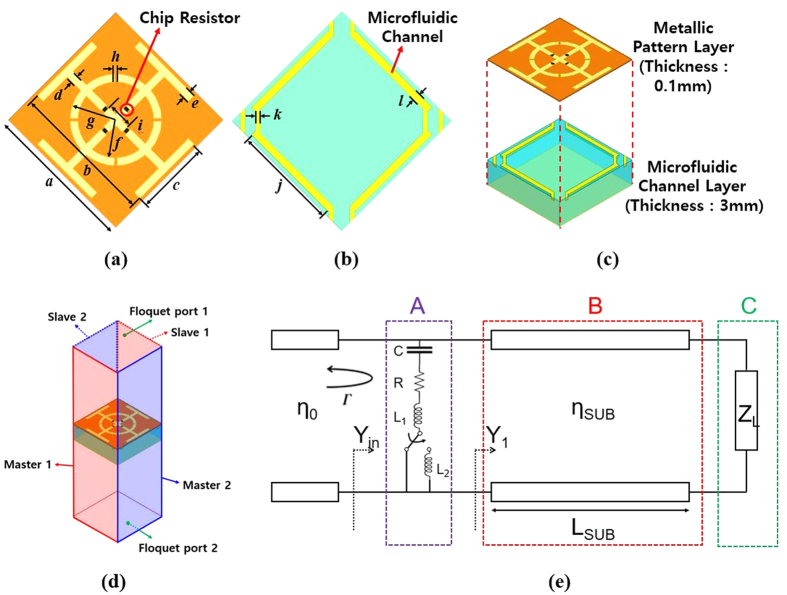
The unit cell of the proposed absorber: a = 10 mm, b = 9.5 mm, c = 5 mm, d = 0.5 mm, e = 0.5 mm, f = 2.5 mm, g = 3 mm, h = 0.2 mm, i = 1.74 mm, j = 7 mm, k = 0.3 mm, l = 0.5 mm. (**a**) JC resonator with slotted circular ring and chip resistors. (**b**) Microfluidic channels. (**c**) Three-dimensional view of the unit cell. (**d**) Boundary conditions and excitations for a periodic structure. (**e**) Transmission line model of the proposed absorber.

The unit cell of the proposed absorber is illustrated in Fig. 1(a). The unit cell is composed of a JC resonator with slotted circular ring—a symmetric and simple design. In order to increase the bandwidth, four 100 Ω chip resistors are added. Figure 1(b) shows the design of the microfluidic channels. In order to switch the absorption frequency band, the microfluidic channel is located just below the outermost arm of the JC resonator. In addition, each microfluidic channel is designed as a single line, therefore, it is easy to inject or remove EGaIn to or from the microfluidic channel. The JC resonator can generate electric and magnetic resonance. Capacitance can be generated from a slot in the circular ring and the gap between adjacent unit cells. Inductance can be generated from the crossed strip and outermost arms. It is easy to change a frequency by varying inductance of the JC resonator.

Figure 1(c) shows a three-dimensional view of the proposed absorber. The proposed absorber consists of two layers. The top layer has the JC resonator pattern and chip resistors on a 0.1-mm thick FPCB, and the bottom layer has the microfluidic channels on a 3-mm thick PDMS substrate. The bottom side of the unit cell is covered with copper sheet to prevent transmission. When EGaIn is injected into the channels, the EGaIn acts as a part of the JC resonator even though the two do not directly touch each other. Therefore, the inductance can be increased by injecting liquid metal under the outermost arms of the JC resonator. This is due to the strong coupling of the incident EM wave. Therefore, by injecting EGaIn into the channels, the proposed absorber has an altered EM response, resulting in a shift of the absorption frequency. Figure 1(d) shows the boundary conditions and excitations for EM simulation.

In order to explain the switching operation and performances of the proposed absorber, the transmission line model is introduced as shown in Fig. 1(e). It consists of three parts of A, B, and C. The load impedance (Z_L_) in the part C represents the ground plane. The top pattern can be represented as a series connection of resistance (R), inductance (L), and capacitance (C) in the part A. Especially, the inductance is L_1_ with empty channels. When liquid metal is injected into the channels, the total inductance becomes L_1_ + L_2_ because of the extended conductive traces. The finite length of the transmission line (L_SUB_) in the part B represents PDMS materials with intrinsic impedance of η_SUB_.

From the transmission line model^11^, the input admittance (Y_in_) of the proposed absorber is given by


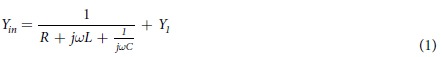



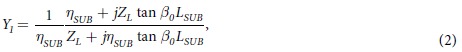


where β_0_ and η_0_ are phase constant and intrinsic impedance of free space, respectively.

The reflection coefficient is given by


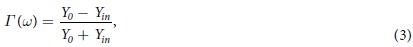


where Y_0_ is the admittance of free space.

It is well known from Eq. (3) that zero reflection coefficient is achieved when Y_0_ and Y_in_ are equal.

**Figure 2:**
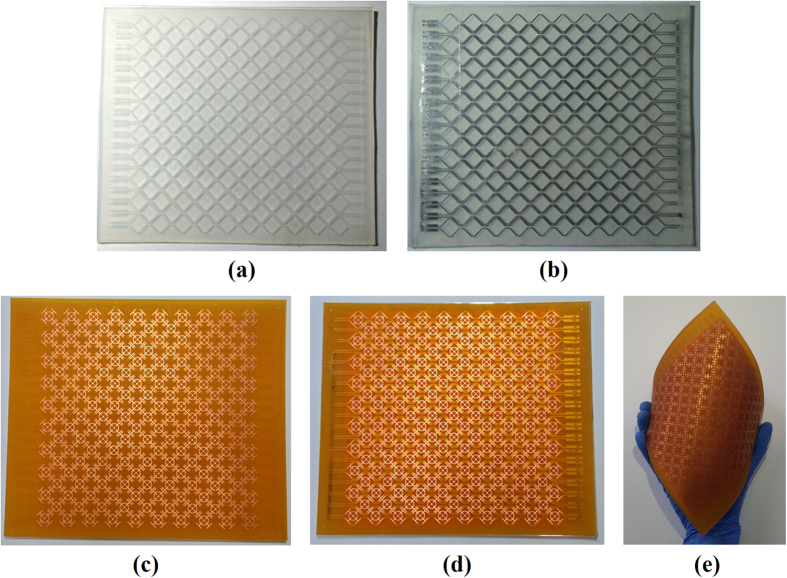
Fabricated microfluidic channel: (**a**) Empty state. (**b**) EGaIn filled state. Fabricated prototype of the proposed absorber with (**c**) empty channels, (**d**) EGaIn-filled channels and (**e**) folded sample.

In order to experimentally verify the performance of the proposed absorber, the microfluidic channels, including inlet and outlet, were fabricated on a 17.5 cm × 15 cm PDMS substrate as shown in Fig. 2(a). The thickness, relative permittivity, and dielectric loss of PDMS were 3 mm, 3, and 0.065, respectively. The microfluidic channels were realized by using a laser etching technique, and the channel depth was 0.3 mm. To attach the FPCB and PDMS substrate, a plasma surface treatment was applied to the PDMS substrate, and then adhesive film (ARcare^®^ 92561) was used as a bonding layer. Figure 2(b) shows the microfluidic channels filled with EGaIn. A final prototype sample, including 181 JC resonators, is shown in Fig. 2(c,d). The JC resonators were realized by using a conventional PCB manufacturing process on 0.1-mm thick FPCB substrate. The 100 Ω chip resistors were soldered by surface mount technology (SMT). The thickness, relative permittivity, and dielectric loss of the FPCB were 0.1-mm, 3, and 0.065, respectively. PDMS and FPCB are flexible, so the proposed absorber was given flexibility.

### Simulated and experimental results

A perfect metamaterial absorber can be achieved by eliminating reflected and transmitted waves. Through effective medium approximation, the metamaterial exhibits frequency dependent permittivity and permeability^18^. Therefore, the intrinsic impedance of the metamaterial, *Z*(*ω*), can be defined by the relation between relative permittivity *ε*_*r*_ and permeability *μ*_*r*_ as shown in the following equation:


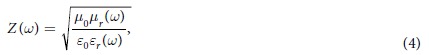


where the *ε*_0_ and *μ*_0_ are the permittivity and permeability of free space, respectively.

By manipulating *ε*_*r*_ and *μ*_*r*_ to have the same value, *Z*(*ω*) becomes the intrinsic impedance of free space *Z*_0_. The reflection coefficient (Γ) under normal incidence is given by


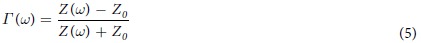


Therefore, the impedances of the metamaterial and of free space are matched, and there are no reflected waves from the surface of the metamaterial.

In addition, the metamaterial has a large imaginary part of the refractive index, *n*, which means the loss component is large. As a result, the metamaterial absorber not only minimizes reflection by matching the impedances of the metamaterial and of free space, but waves transmitted into the metamaterial are dissipated by the large loss component.

The JC resonator generates electric resonance through the capacitive and inductive components of the pattern. A slot in the circular ring and the gap between adjacent unit cells generate capacitance, and the crossed strip generates inductance. The capacitance of the JC resonator can be calculated from its effective dielectric constant, *ε*_*eff*,_ and the length, *l*, of the capacitive gap^25^:


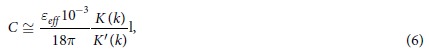


where the *K*(*k*)/*K*′(*k*) is the approximate ratio of the elliptic integrals. From equation (6), it can be seen that the capacitance of the JC resonator can be modified by changing its geometry. As a result, the resonant frequency of the JC resonator can be shifted. In this study, we used microfluidic channels and EGaIn to modify the resonator pattern to change the frequency band.

**Figure 3:**
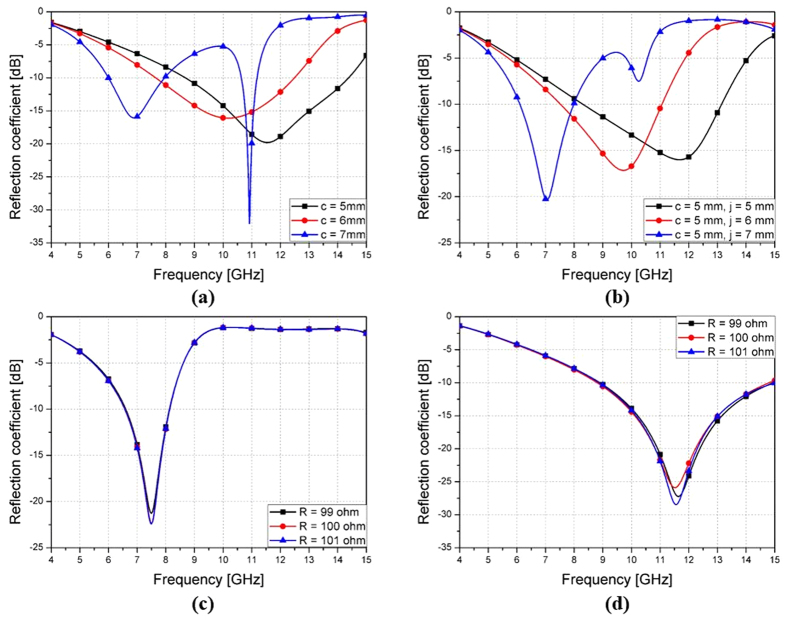
Simulation results for (**a**) different lengths of *c* without the microfluidic channels, (**b**) different lengths of *j* without the microfluidic channel connection, (**c**) EGaIn-filled channels and (**d**) empty channels with ± 1% variation of resistance.

By incorporating chip resistors as additional loss components, the proposed absorber achieved a wideband absorption characteristic. To shift the wideband frequency range, we needed to make a significant change to the values of the capacitive and inductive components. There were several parameters that could be used to change the capacitive and inductive components. The most effective way was to change the length of the outermost arm, *c*, because there was enough margin and because it could minimize the parasitic component generated by the modification. Figure 3(a) shows the simulated reflection coefficients for different lengths of *c* without microfluidic channels. By increasing the length of *c*, the resonant frequency becomes lower. The next step was to include microfluidic channels filled with EGaIn. The microfluidic channels are isolated from the metallic pattern layer by placing them underneath the outermost arms. The simulated reflection coefficients for different lengths of *j* with liquid-metal filled channels are shown in Fig. 3(b). By increasing the length of *j* while keeping c = 5 mm, the resonant frequency becomes lower. When *c* or *j* are longer than 7 mm, the additional resonance occurs in high frequencies due to coupling between outermost arms. It shows similar 10 dB bandwidths compared to Fig. 3(a), due to incident wave coupling, even though the EGaIn and the JC resonator pattern do not directly touch each other. In general, a chip resistor has 1% tolerance of its resistance. In order to see the effect of this tolerance, the absorptivity with empty and liquid metal-filled channels are simulated with ± 1% variation of resistance. The absorptivity with liquid metal-filled channels is not affected by variation of resistance as shown in Fig. 3(c). The absorptivity with empty channel is slightly changed as shown in Fig. 3(d).

With regard to injecting and removing EGaIn, the channel design with connections is shown in Fig. 1(b). To minimize unwanted parasitic components, the connection width *k* needed to be narrow. However, by the Young-Laplace equation, the minimum pressure to generate flow was inversely proportional to the cross sectional dimensions of the microfluidic channel^26^. Taking into account the required pressure and the viscosity of EGaIn (twice that of water)^27^, the width of *k* was determined.

**Figure 4:**
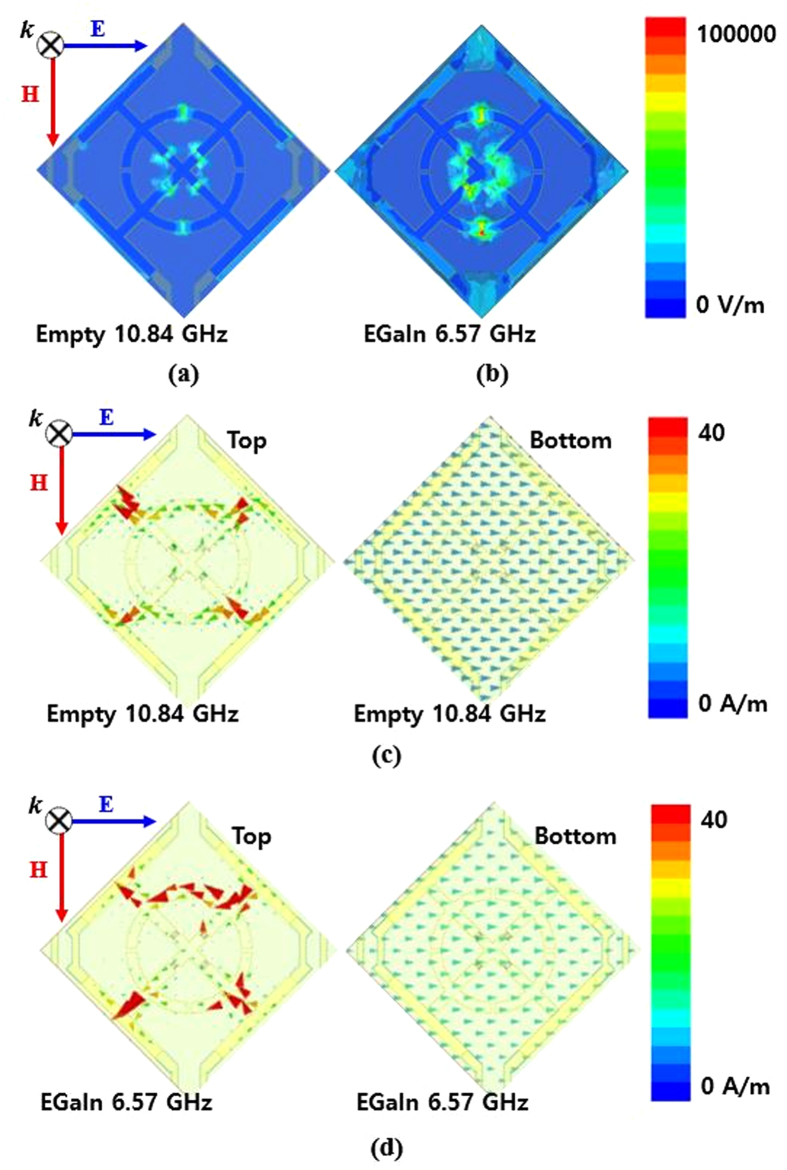
Simulated electric field distribution for (**a**) empty channels and (**b**) EGaIn-filled channels. Simulated vector current distribution for (**c**) empty channels, and (**d**) EGaIn-filled channels.

In order to verify the electric and magnetic resonances of the proposed absorber, the electric field distributions and vector current distributions were plotted. When the channel was in its empty state and the frequency was set to 10.84 GHz, most of the electric fields were generated at the slots in the circular ring and at the slots for the chip resistors, as shown in Fig. 4(a). When the channel was filled with EGaIn, additional fields were generated around the outermost arms. Therefore, the resonant frequency shifted to 6.57 GHz, as shown in Fig. 4(b). In addition, magnetic resonances were generated by anti-parallel currents at the top and bottom sides of the proposed absorber, as shown in Fig. 4(c,d).

**Figure 5:**
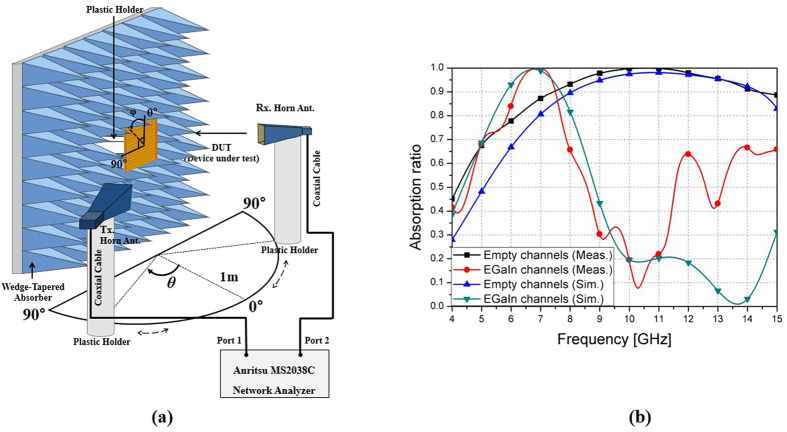
(**a**) Illustration of the bistatic radar cross section measurement setup, (**b**) Simulated and measured absorption ratios for empty channels and EGaIn-filled channels under normal incidence.

To demonstrate the performance of the proposed absorber, a bistatic RCS measurement setup was used to measure the scattering parameter^11^. An illustration of the measurement setup is shown in Fig. 5(a). The absorption ratio, *A*(*ω*), can be calculated by the reflectance, *R*(*ω*), and the transmittance, *T*(*ω*), as given by:


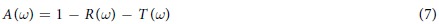


However, the bottom side of the proposed absorber was fully covered with copper sheet, so the EM wave could not pass through the proposed absorber. Therefore, we did not need to consider the transmission coefficient, and could obtain the absorption ratio by measuring only reflection coefficients. Before measuring reflection coefficients of the absorber prototype, for calibration purposes, we first measured the reflection coefficient of a copper plate with the same size as that of the fabricated absorber prototype. After measuring the reflection coefficient of a copper plate, we set its reflection coefficient as -1 28.

Figure 5(b) shows simulated and measured absorption ratios for a normally-incident EM wave for both the empty state and the EGaIn-filled state of the microfluidic channels. The proposed absorber showed an over 90% absorption ratio from 7.43 GHz to 14.34 GHz in the empty state. When the channels were filled with EGaIn, the proposed absorber showed an over 90% absorption rate from 5.62 GHz to 7.3 GHz. It takes 5 minutes to inject EGaIn in to all microfluidic channels by a syringe. The injection time can be significantly by a pneumatic micropump because its maximum fluid pumping rate is 100 μL/min^29^. Although EGaIn is repeatedly filled and removed 10 times, the performances are not changed.

**Figure 6:**
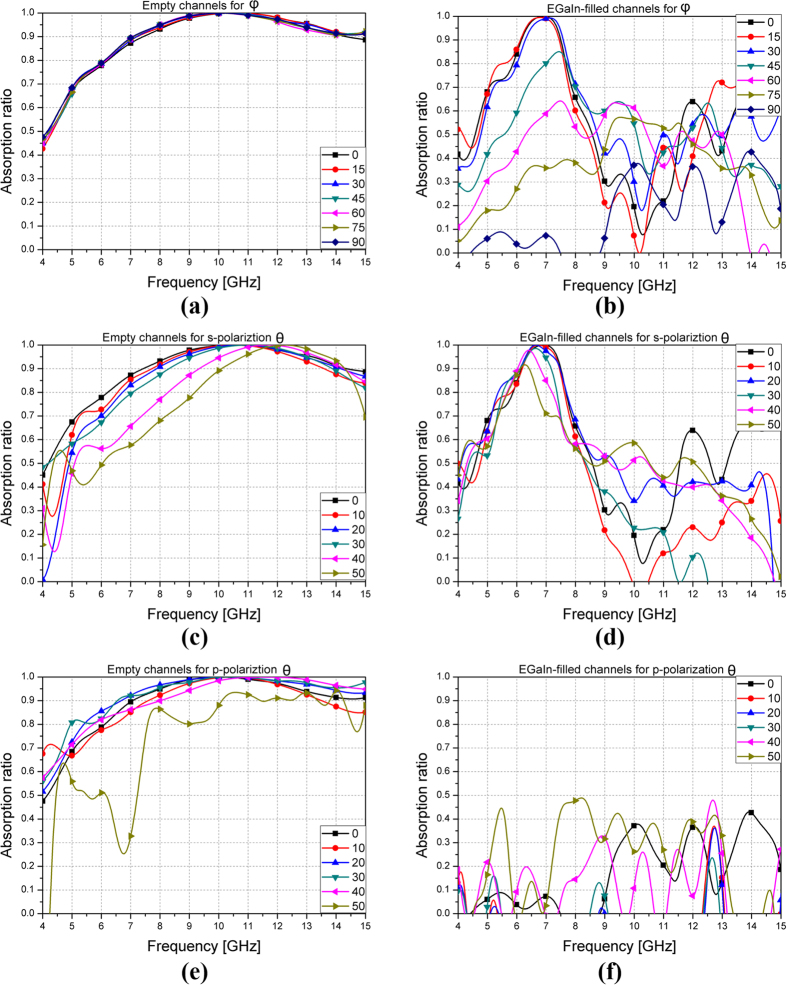
Measurement results at different polarization (*φ*) and incident (*θ*) angles for s-and p-polarization: (**a**) Empty channels and (**b**) EGaIn-filled channels for *φ*. (**c**) Empty channels and (**d**) EGaIn-filled channels for s-polarization *θ*. (**e**) Empty channels and (**f**) EGaIn-filled channels for p-polarization *θ*.

We also verified polarization and incident angle dependence of the proposed absorber though measurements. The prototype sample was rotated by angle *φ* to verify the polarization angle dependence. Figure 6(a) shows the results for different angles *φ* when the channel was empty. The 90% absorption bandwidth of the proposed absorber is maintained from 7.43 GHz to 14.34 GHz at all angles *φ*. For the EGaIn-filled channel, the absorption ratio changed drastically when the angle *φ* changed, as shown in Fig. 6(b). This was due to the asymmetric channel design, particularly the design of the channel connections.

The absorption ratio of the fabricated prototype was measured for oblique incident waves polarized in s- and p-polarization. Figure 6(c,d) show the results for s-polarization waves at different incident angles. For the s-polarization, the two horn antennas were placed at angle *θ* to satisfy Snell’s law, and the electric field direction of the antennas was perpendicular to the ground. The performance with empty channels and with EGaIn filled channels was lower at 50°. The p-polarization measurement setup was the same as for the s-polarization except that the electric field direction of the antennas was parallel to the ground. Figure 6(e,f) show the results for the p-polarization at different incident angles. The empty state results were similar to the s-polarization, but when the channels were filled with EGaIn, the prototype absorber could not act as an absorber, due to the asymmetry of the microfluidic channel design. This problem can be solved by horizontal and vertical symmetric unit cells with liquid metal-filled channels.

**Figure 7:**
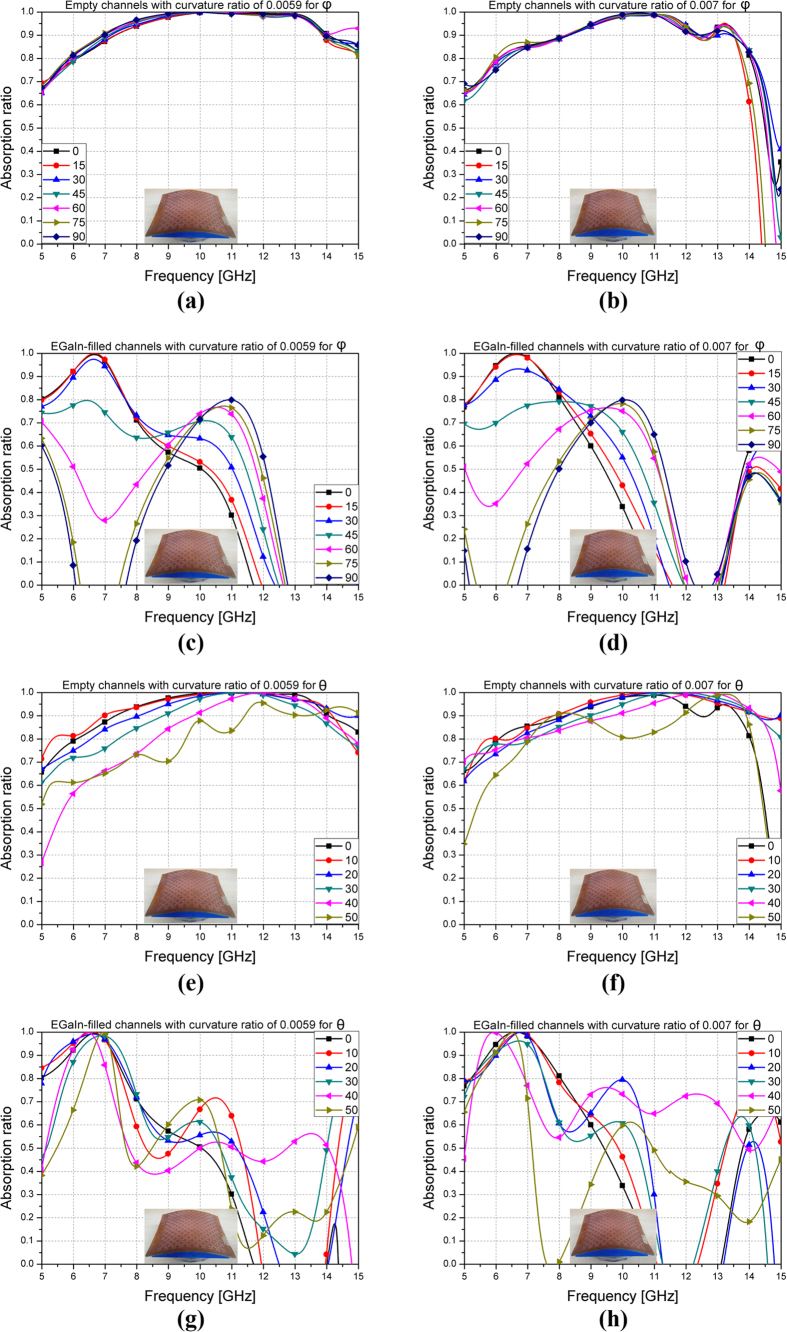
Measurement results at different polarization (φ) and incident (θ) angles on curved surface: Empty channels for different φ with curvature ratio (**a**) 0.0059 and (**b**) 0.007. EGaIn-filled channels for different φ with curvature ratio (**c**) 0.0059 and (**d**) 0.007. Empty channels for different θ with curvature ratio (**e**) 0.0059 and (**f**) 0.007. EGaIn-filled channels for different θ with curvature ratio (**g**) 0.0059 and (**h**) 0.007.

The performances of the proposed absorber on curved surface are investigated in Fig. 7. Two cylindrical surfaces with curvature ratio (CR) of 0.0059 and 0.007 are used as shown in the inset of Fig. 7. Figure 7(a,b) show the measured absorption ratio with empty channels for φ with curvature ratio of 0.0059 and 0.007, respectively. Figure 7(c,d) show the measured absorption ratio with EGaIn-filled channels for φ with curvature ratio of 0.0059 and 0.007, respectively. Figure 7(e,f) show the measured absorption ratio with empty channels for *θ* with curvature ratio of 0.0059 and 0.007, respectively. Figure 7(g,h) show the measured absorption ratio with EGaIn-filled channels for *θ* with curvature ratio of 0.0059 and 0.007, respectively. S-polarization is used in Fig. 7(e–h). Similar to flat surface, the absorption ratio with empty channels is almost not changed although φ is varied as shown in Fig. 7(a,b) because of the symmetric unit cell. However, the absorption ratio with EGaIn-filled channels is changed at different φ as shown in Fig. 7(c,d) because of the asymmetric unit cell. For oblique incidence on the proposed sample with empty channels and curvature ratio of 0.0059 and 0.007, it is observed from Fig. 7(e,f) that the absorption frequency increases and bandwidth decreases with higher θ. For oblique incidence on the proposed sample with EGaIn channels, it is observed from Fig. 7(g,h) that the absorption frequency is shifted and bandwidth decreases with higher θ.

Table 1 lists the 90% absorption bandwidth of the fabricated absorber on curved surface with other CR from 0 to 0.0167. The absorptivity under normal incidence are measured at CR = 0, 0.0059, 0.007, 0.01, and 0.0167. Compared to flat surface (CR = 0), the 90% bandwidth becomes narrower with higher CR. Nevertheless, the absorptivity with empty channels is kept higher than 90% for X-band until CR is 0.007. For the EGaIn-filled channels, the frequency increases with higher CR while keeping larger than 20% bandwidth until CR is 0.01.

**Figure 8:**
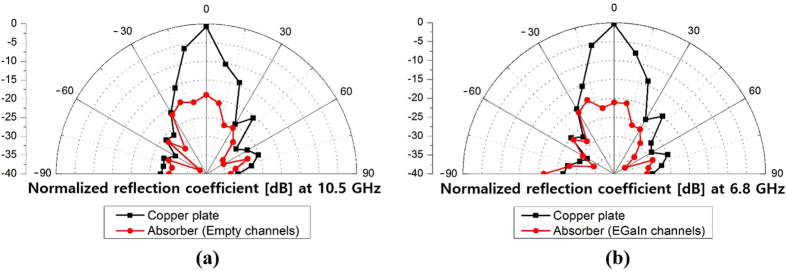
Bistatic RCS measurement results at (**a**) 10.5 GHz for empty channels and (**b**) 6.8 GHz for EGaIn-filled channels.

The bistatic radar cross section (RCS) was also verified through measurement. To measure the bistatic RCS, the transmitting antenna was fixed normal to the prototype sample (*θ* = 0°), and the receiving antenna was rotated from −90° to + 90°. The results at 10.5 GHz for empty channels, and at 6.8 GHz for EGaIn-filled channels, are plotted in Fig. 8. The RCS graphs of the proposed absorber are normalized to the RCS of a copper plate. The normalized RCS for both the empty-channels state and the EGaIn filled-channels state of the prototype absorber was 20 dB lower than that of the copper plate for normally incident waves. In addition, no scattering from the proposed absorber was observed in other directions, and most power was absorbed.

## Discussion

In conclusion, we proposed a flexible wideband-switchable metamaterial absorber using EGaIn. The proposed absorber consisted of a metallic pattern layer and a microfluidic channel layer, with the layers bonded by an adhesive laminating film. In order to demonstrate its performance, 181 unit cells were fabricated. A conventional PCB manufacturing process was adopted to realize the resonator patterns, and SMT was used to solder the chip resistors. The proposed absorber showed over 90% absorption rate from 7.43 GHz to 14.34 GHz in the empty-channels state. When the channels were filled with EGaIn, the proposed absorber showed an over 90% absorption rate from 5.62 GHz to 7.3 GHz. The materials used for the substrate gave the proposed absorber much greater flexibility than a conventional PCB- based metamaterial absorber. Although absorption bandwidth is decreased with higher curvature surface, the proposed absorber keeps higher than 90% absorptivity in the X-band until curvature ratio is 0.007. Furthermore, the proposed absorber used microfluidics for the switching component, so it did not require continuous power consumption while in use.

## Methods

### Simulation

The proposed absorber was simulated by applying the finite-element method (FEM), using the ANSYS high-frequency structure simulator (HFSS). Master/slave pairs are applied as the boundary condition to model a periodic structure. When using a master and slave pair, the E-field on one surface, the slave surface is forced to match the E-field on another, the master surface, to within a phase difference. In addition, Floquet ports are used for excitation of a periodic structure. Due to the finite size of the fabricated prototype sample, there was a difference between the measured and the simulated results, as shown in Fig. 5(b). In addition, the packaging of the chip resistors and the presence of solder generated unexpected scattering at the surface of the proposed absorber. These were additional reasons for the difference.

### Measurement

An Anritsu MS2038C vector network analyser was used for measuring scattering parameters. To prevent unwanted signals, the fabricated prototype absorber was surrounded by a wedge-tapered absorber. In addition, a time gating function of the vector network analyser was employed to measure only the reflected signals from the prototype absorber. To satisfy the far field condition, two standard gain horn antennas were place 1 m away from the prototype absorber. For oblique incidence, two standard-gain horn antennas are used as the transmitting and receiving antennas. For normal incidence, a single antenna is enough to measure reflection coefficient. We established a measurement reference by measuring the reflection coefficient of a copper plate that had the same size as the prototype absorber.

## Additional Information

**How to cite this article**: Kim, H. K. *et al*. Wideband-Switchable Metamaterial Absorber Using Injected Liquid Metal. *Sci. Rep*. **6**, 31823; doi: 10.1038/srep31823 (2016).

## Figures and Tables

**Table 1:** Summary of 90% absorption bandwidth of the fabricated absorber with different curvature ratio.

CR	90% Absorption Bandwidth under Normal Incidence	
	Empty channels	EGaIn-filled channels
0 (Flat)	7.43–14.34 GHz (63.5%)	5.62–7.30 GHz (26.1%)
0.0059	7.33–14.06 GHz (62.9%)	5.86–7.28 GHz (21.6%)
0.007	8.20–13.65 GHz (49.9%)	5.76–7.54 GHz (26.7%)
0.01	8.01–11.05 GHz (35.8%)	5.65–7.95 GHz (33.8%)
0.0167	9.11–11.24 GHz (20.9%)	6.45–7.50 GHz (14.9%)
